# About Functional Foods: The Probiotics and Prebiotics State of Art

**DOI:** 10.3390/antibiotics12040635

**Published:** 2023-03-23

**Authors:** Andrea Ballini, Ioannis Alexandros Charitos, Stefania Cantore, Skender Topi, Lucrezia Bottalico, Luigi Santacroce

**Affiliations:** 1 Department of Precision Medicine, University of Campania “Luigi Vanvitelli”, 80138 Naples, Italy; andrea.ballini@me.com; 2 National Poisoning Center, Emergency/Urgent Department, Riuniti University Hospital of Foggia, 71122 Foggia, Italy; alexanestesia@hotmail.com; 3 Independent Researcher, Regional Dental Community Service “Sorriso & Benessere-Ricerca e Clinica”, 70129 Bari, Italy; 4 Department of Clinical Disciplines, University of Elbasan, 3001 Elbasan, Albania; skender.topi@uniel.edu.al (S.T.); bottalico.lu@gmail.com (L.B.); 5 Microbiology and Virology Unit, Department of Interdisciplinary Medicine, University of Bari “Aldo Moro”, 70124 Bari, Italy; luigi.santacroce@uniba.it

**Keywords:** intestinal microbiota, dysbiosis, functional foods, probiotics, prebiotics, microencapsulation

## Abstract

Poor diet, obesity and a sedentary lifestyle have a significant impact on natural microbiota disorders; specifically, the intestinal one. This in turn can lead to a multitude of organ dysfunctions. The gut microbiota contains more than 500 species of bacteria and constitutes 95% of the total number of cells in the human body, thus contributing significantly to the host’s resistance to infectious diseases. Nowadays, consumers have turned to purchased foods, especially those containing probiotic bacteria or prebiotics, that constitute some of the functional food market, which is constantly expanding. Indeed, there are many products available that incorporate probiotics, such as yogurt, cheese, juices, jams, cookies, salami sausages, mayonnaise, nutritional supplements, etc. The probiotics are microorganisms that, when taken in sufficient amounts, contribute positively to the health of the host and are the focus of interest for both scientific studies and commercial companies. Thus, in the last decade, the introduction of DNA sequencing technologies with subsequent bioinformatics processing contributes to the in-depth characterization of the vast biodiversity of the gut microbiota, their composition, their connection with the physiological function—known as homeostasis—of the human organism, and their involvement in several diseases. Therefore, in this study, we highlighted the extensive investigation of current scientific research for the association of those types of functional foods containing probiotics and prebiotics in the diet and the composition of the intestinal microbiota. As a result, this study can form the foundation for a new research path based on reliable data from the literature, acting a guide in the continuous effort to monitor the rapid developments in this field.

## 1. Introduction

Nowadays, consumers have turned to the purchase of functional foods that could prevent the onset of dysbiosis of the human microbiota and, therefore, of diseases connected to it. A human’s daily nutrition in the context of diet has an impact on his health and requires the presence of foods with more benefits than the simple supply of energy, mineral salts, trace elements and vitamins—the so-called functional foods [1,2]. More specifically, the concept of promoting functional foods began in Japan in 1984 because studies had demonstrated the connection between nutrition, taste satisfaction, activation of physiological systems (such as immunity) and food fortification. Subsequently, research on the design of these products moved to Europe and America [3]. A characteristic of these foods is that various definitions exist. An “official” definition of functional foods is as follows: a food can be defined as functional if, together with its basic nutritional action, it has a beneficial effect on one or more physiological functions, such as to improve general health and/or to reduce the risk of developing disease. Therefore, they are foods that, in addition to nutritional value, offer health benefits. These foods have nutrient-rich ingredients (such as fruits and vegetables) but can also be enriched with vitamins, minerals, probiotics, prebiotics, and fibers [4,5]. The form of food and the required intake should be intended for different purposes; therefore, they cannot be available in pill or capsule form. In simple words, they are foods that fulfill the body’s nutritional needs and, at the same time, have a beneficial effect on the body, if consumed in reasonable quantities and always according to the principles of proper nutrition [6]. Probiotics are microorganisms which, if taken in sufficient quantities, contribute positively to the health of the host and are the focus of both scientific studies and commercial companies. The market in the field of functional foods and, specifically, those containing probiotic bacteria, which constitute 60% of all functional foods, is constantly expanding. Nowadays, probiotic products are steadily expanding their market share; this is happening mainly in the developed countries of Europe, Japan, Australia, and America [7]. Several alimentary products are incorporating or encapsulating probiotic strains and are available in juices, types of yoghurts, cheeses, jams, biscuits and food supplements. Encapsulation offers many benefits to microorganisms, such as vitality, functionality, strength, and protection. Some of the probiotic foods contain additional bioactive ingredients such as stanols and plant sterols that lower cholesterol levels [8,9]. Although at present there is no official statement on what probiotics are, in recent years many authors have tried to give their own definitions. For the development of probiotics with desirable actions, it is necessary to know some parameters and in particular the conditions that prevail in the gastrointestinal tract. The purpose of this study is to give an snapshot of what has been discovered about probiotics and prebiotics in functional foods. Therefore, we have highlighted the extensive investigation of current scientific research regarding the association of the types of functional foods containing probiotics and prebiotics in the diet, as well as the composition of the intestinal microbiota. This work can form the foundation for a new research path based on reliable data from the literature, and it can act as a guide in the continuous effort to monitor the rapid developments in this field.

## 2. Probiotics and Prebiotics Concepts

**Figure 1:**
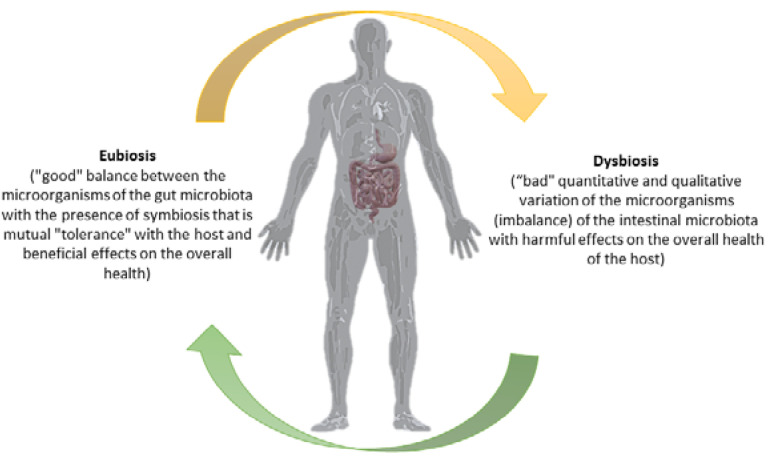
The gut microbiota/host axis depends on the favorable balance between the microorganisms that constitute it. Credits: Original figure by I.A. Charitos.

The Nobel Prize winner for Medicine in 1908, Elie Metchnikoff, argued that the cause of aging is toxin released by the decaying of certain bacteria in the intestine, or, by the degradation of components through the release of proteolytic enzymes from *Clostridium* spp. [10]. In fact, he stated that “*the dependence of friendly bacteria on food allows measures to be taken to modify the microbial composition of our body and thus replace the harmful ones*”. Metchnikoff’s scientific hypothesis on fermented milk obtained from *Bacillus bulgaricus* (actually called *Lactobacillus delbrueckii subsp. Bulgaricus)* laid the foundation for the development of the first dairy industry [10,11]. In addition to the application to whey and food, probiotics began to be used to improve the health of patients. In 1989, Fuller defined a probiotic as a dietary supplement with live microbes which has a positive effect on the host by improving the microbial balance of the host’s gut. Subsequently, in 1991 he defined probiotics as single or mixed cultures of live microorganisms that have a beneficial effect when administered to humans or animals and contribute to the improvement of the properties of their acquired endogenous microbial presence [12]. In 1998, a group of scientists proposed that probiotics are food components composed of living microorganisms that have a beneficial effect on health [13]. One of the simplest and most accepted explanations was that proposed by a committee of experts composed of members of the Food and Agriculture Organization of the United Nations and the World Health Organization which states: “*Probiotics are microorganisms which, when administered sufficient quantities, confer a beneficial action on the health of the patient*”. Thus, they prevent and aid the patient in avoiding the dysbiosis and lead to the gut microbiota’s eubiosis (Figure 1).

Recently, probiotics have been characterized as live microorganisms that show resistance to gastric, biliary and pancreatic secretions, adhere to epithelial cells, and colonize the human intestine. Therefore, probiotics are bacteria which are beneficial to health, which now also have clinical effects [14]. Probiotic microorganisms must usually be of human origin, and therefore safe, and they must maintain their vitality, both during the technological processes that the food undergoes and during their passage through the gastrointestinal tract [11,12,13,14,15]. Equally desirable are the possibilities of their immobilization in the intestinal epithelium, the competitive action against pathogenic microorganisms, and their resistance to antibiotic substances. Probiotic bacteria are kept alive both in freeze-dried form and when injected into fermented products (Table 1) [10].

The most used probiotic species are from *Lactobacillaceae* family, *Bifidobacteria* spp. and yeasts such as *Saccharomyces boulardii* [11,12]. 

Probiotics can be taken through specific formulations (supplements), through dairy functional foods (such as yoghurt, cheese, ice cream and other) and through non-dairy products [14]. Since probiotics are sensitive to environmental factors, such as the inhospitable environment of the gastrointestinal tract, various techniques are used to protect them; mainly the micro-encapsulation technique is used, so that they maintain the best performance. In general, various species from *Lactobacillaceae* family (such as *Lactobacillus acidophilus*, *Lacticaseibacillus casei, Lacticaseibacillus rhamnosus.* and *Lactobacillus helveticus)* have been extensively studied for the prevention of certain health disturbances, such as the non-communicable diseases (NCDs) [15]. However, some tests have shown adverse effects associated with the administration of probiotics, as is common with any preparation. Most often, side effects are mild and include symptoms such as nausea, indigestion and abdominal discomfort (such as flatulence, and constipation). Less commonly, infections can develop; the most serious side effects that have been reported are endocarditis and septicemia [13]. A positive attitude toward probiotics has been observed by healthcare professionals, and also by consumers, as they are not considered medicines. It should be noted, however, that the safety and efficacy of probiotics must be determined by considering the quantity and dosage of the probiotics, the characteristics of the consumer (including the research of the metabolic profile of his intestinal microbiota) and the reason for the intake of probiotics. These reasons make it necessary to research an individualized probiotic treatment for each individual [14,15,16]. 

Prebiotics are products of food digestion that have a positive effect on the health of the host. Thus, their purpose is to modify the composition of the intestinal microbiota, in order to favor the growth of probiotic bacteria and inhibit the growth of unfriendly or unwanted microorganisms [16,17]. In 2008, the FAO (Food and Agriculture Organization of the United Nations) defined prebiotics as “*a non-vital food ingredient that benefits the health of the host associated with microbiological modulation*” [18].

Prebiotics are functional alimentary ingredients that are found naturally in vegetable foods or can be produced by synthetic production through enzymatic conversion of sugars [13]. These alimentary compounds are generally structures of carbohydrates or soluble alimentary fibers, which are selectively metabolized by the human microbiota. The most used prebiotics in Europe are galattooligosaccarides (GOS) and inulin derivatives, such as fructooligosaccarides (FOS) [16]. The GOS derived from lactose is found in human milk and vaccines, but are also present as additives in many other foods, such as cereals and dairy products [19]. GOS favors the proliferation strains from the *Lactobacillaceae* and *Bifidobacteriaceae* families, which are highly beneficial for the host’s health [16]. Moreover, these prebiotics can prevent infection by pathogenic microorganisms, because they can structurally imitate their binding sites and prevent their adhesion to epithelial cells [20]. FOS are fructans, hydrolytic derivatives of inulin, with a small number of fructose monomers. They are found in high percentages in plant foods, such as onion, asparagus, wheat and artichoke. [19,20,21]. Prebiotics, such as cellulose, lignin and oligosaccharides, present in foods such as raw oats, soybeans and chicory roots, must resist gastric acids and be able to reach the large intestine in order to be fermented by the intestinal microbiota, which favors the development of beneficial intestinal species [12]. Recent studies have shown that non-digestible carbohydrates, such as resistant starch and fiber, are not metabolized in the small intestine and for this reason they can reach the large intestine, where they are fermented by the intestinal microbiome [14]. In this context, non-digestible carbohydrates can potentially act as prebiotics, stimulating the growth of some species that contribute to the health of the host [22]. 

Several studies have shown that prebiotics are able to influence metabolic and immune factors such as IL-6, insulin resistance and the amount of glucose in the blood. These data indicate that the intestinal microbiota is able to regulate the host’s metabolism and immune response as a function of the diet, thus contributing to the maintenance of the host’s state of health [23]. 

Prebiotics, therefore, provide benefits to the host, including strengthening the integrity of the intestinal mucosal barrier, increasing host mucosal immunity, lowering pH and the production of Short Chain Fatty Acids (SCFAs) and inhibiting the growth of pathogenic microorganisms [24]. The use of prebiotics also plays a role in the treatment of obesity. The presence of prebiotics in the gut is associated with the production of both protective mucus and SCFAs, as well as with the production of anti-inflammatory cytokines. Furthermore, it is associated with the secretion of satiety hormones and thus prevents overeating. [25]. Fructose-containing oligosaccharides are mainly used as prebiotic substances. Thus, they have a favorable effect on the growth of probiotic bacteria by limiting the surface area that would otherwise be occupied by pathogenic microorganisms (such as *Escherichia coli*) [25]. It is also possible to use other oligomers of specific sugars, such as lactulose, soy and maltose, as well as oligosaccharides containing xylose, mannose, and galactose. Finally, honey is considered a prebiotic food. Prebiotic oligosaccharides can be obtained by (a) isolation from plant raw materials, (b) biotechnological production or enzymatic synthesis and (c) enzymatic hydrolysis of polysaccharides. Many prebiotic oligosaccharides are produced on an industrial scale and are commercially available [16]. Indeed, β(1-2) fructans (such as inulin and/or fructo-oligosaccharides) are the best studied prebiotics and are present in various foods (such as leeks, onions, garlic, artichokes, asparagus, shallots, bananas and wheat) [22]. Their average consumption, in an ordinary diet, is estimated at a few grams per day and they have been recognized as dietary fibers in most countries. Industrially, inulin is derived from hot water extraction of radish roots, followed by refining and spray drying. High inulin (>90%) is commercially available as a white powder. Like oligofructose, it is obtained industrially by partial enzymatic hydrolysis of inulin using a special endoinulase [20]. Furthermore, fructooligosaccharides (FOS) can also be produced by transfructosylation of sucrose. Commercial preparations of oligofructose include various contents, up to 95%, and are in the form of white powders and sticky syrups [26]. As for soy oligosaccharides, they are obtained by direct extraction and purification from soy milk whey, a by-product of soy protein concentrate production. They are marketed as a syrup containing 6% raffinose and 18% stachyose, as well as sucrose, glucose and fructose (52%) [26]. Some galactooligosaccharides (GOS) components have been reported to occur naturally in human milk at an amount of about 3mg/L [16]. Industrially transgalactosylated oligosaccharides are produced from the synthesis of lactose by using a β-galactosidase [18]. In practice, the combined use of probiotics and prebiotic ingredients is usually applied due to their synergistic action in foods. In this way, symbiotic products are created that benefit the consumer through the survival and establishment of selected live microorganisms in the digestive system [26,27]. 

## 3. The Microbiota of the Gastrointestinal Tract

Microbial communities are found on a variety of environmental surfaces (such as gastrointestinal, skin, genitourinary, upper and lower airway tract, etc.). The human microbiota and diet environment, ethnicity, genotype, gender and, in general, the individual’s lifestyle (sedentary life, exercise, smoking, abuse substances, chemical compounds such as bisphenol A, etc.), are factors that interact with each other and influence health and the development of diseases [28,29]. Indeed, the gut microbiota dysbiosis induces metabolic dysfunction and can activate a chronic irregular immune response. This results in the development of metabolic diseases, such as obesity, and the inflammatory response in various body tissues involving various systems, such as the CNS. In terms of gender, differences have been observed in the prevalence of some diseases. Therefore, it seems that some different functions (such as hormones, etc.) that characterize the gender, in combination with the gut microbiota, play a role in manifestation and development of these diseases [30]. 

After observing the structure and functions of the gastrointestinal tract, we can conclude that there is a difference both in the composition and in the function of the microbiota that colonizes it. Indeed, the factors that are influencing the survival and activity of microbiota and probiotic microorganisms are the pH value, intestinal secretions (such as the pancreatic enzymes), place and residence time of probiotics strains in the gastrointestinal tract. The low pH of the stomach serves for the initial stages of digestion, as enzymes in the stomach and pancreas are activated which aid in digestion by breaking down macromolecules such as proteins [31,32]. Additionally, the epithelial glands of the gastrointestinal tract secrete pH-neutralizing alkaline mucus to protect the small intestine. In the small intestine, the transit time of intestinal contents tends to keep the bacterial load below 10^4^ CFU/mL and 10^6^ CFU/mL, and the large intestine is the most populated with 10^11^ to 10^12^ CFU/mL. In fact, the colon is the most hospitable habitat for various microbial populations due to its stable alkaline pH environment [32,33,34,35,36,37,38]. In addition to the transit time, the intestine maintains the balance of its microbiota thanks to the secretions and the low pH that prevails in the stomach. In the stomach, among the bacteria that manage to colonize it, there are *Helicobacter pylori (H. pylori)* strains (1-]type I is highly virulent, with the presence of an 2] intermediate strains, and 3] type (II strains with, reduced virulence) [39,40]. This bacterium uses the hydrolysis products of urea, ammonia and carbon dioxide, and can be found free or attached to the cells. Some strains act as symbiotics while others are pathogenic. Due to the emergence of resistant strains, although they can be treated with antibiotics, an infection in the stomach can persist for a long period of time. Many individuals infected with this bacterium have developed acute gastritis and chronic infection associated with atrophic gastritis and nutritional deficiencies (such as low iron and vitamin B_12_), as well as the development of ulcers and stomach cancer [41,42]. However, recent research has shown that their effect on human health is more complex. Surprisingly, some of these misunderstood bacterial species appear to offer protection against certain diseases, such as asthma and the gastroesophageal reflux disease [43]. Furthermore, these strains affect the metabolic functions of the body and protect against esophageal cancer. Finally, *H. pylori* affects the production of hormones (such as ghrelin and leptin) that regulate appetite and fat storage. Absorption of most nutrients takes place in the small intestine, and if the number of bacteria were disproportionately high, the absorption of nutrients by the body would be hindered [44]. 

In the proximal small intestine, there are strictly anaerobic bacteria that survive under hypoxic conditions, but also facultative anaerobic microorganisms that survive with and without oxygen. The structure of the small intestine has several features that support nutrient absorption while also being an ecosystem for various microorganisms also find in other mucosal tissues [45]. The dominant genera of bacteria are from *Bacteroidota, Bacillota* and *Actinomycetota* phyla. The most populous of these three phyla is the *Bacteroidota,* which are quite versatile in their environment, and, thanks to their high adaptability to different pH values and ability to digest both proteins and carbohydrates, they can settle in different parts of the gastrointestinal tract. Some genera of the *Bacteroidota* phyla are gram-negative and make up 25% of bacteria. These genera aid in the digestion of food to produce metabolites, which are beneficial to the host, and in the removal of toxic bacteria metabolism products [46,47]. However, *Bacteroides* spp. can, under certain conditions, exhibit pathogenic behavior. In fact, *Bacteroides fragilis* (generally commensal), apart from the gut microbiota (in the colon), can cause serious infection if it passes into the bloodstream or surrounding tissue following surgery, disease, or trauma [47]. It is noteworthy that approximately 20% of human cancers are associated with chronic inflammation and persistent infection. Some examples of this association are the chronic inflammatory bowel diseases which are associated with the occurrence of colon cancer [46,47,48]. Indeed, it has been noticed that individuals with increased levels of not only the *Bacteroides* spp., but also *Clostridium* spp. and *Bifidobacterium* spp. in their intestinal microbiota have a higher probability of occurrence of colon cancer; meanwhile, individuals with high concentrations of lactic acid bacteria have a reduced risk of developing this disease [48]. Some *Bacteroides* spp. can use different substances depending on their availability; this is due to the involvement of many genes in starch metabolism. The host organism lacks the appropriate enzymes to degrade complex polysaccharides such as the *Bacteroides thetaiotaomicron,* which produces different enzymes when it senses carbohydrates in the intestinal lumen [49]. This species is involved in the metabolism of two types of carbohydrates: dietary (β-glucans, fructans) and carbohydrates derived from the host organism. The main dietary carbohydrates that constitute a source of nutrients for *B. thetaiotaomicron* are glucans and fructans. These symbiotic bacteria produce various enzymes (such as the endolevanase) responsible for the degradation of these polysaccharides (important prebiotics for these bacteria), which are not metabolized by the body of the host [50,51]. The digestive adaptability of *B. thetaiotaomicron* contributes to the maintenance of intestinal homeostasis by allowing the intestinal microbiota to respond better to dietary changes without altering its qualitative and quantitative microbial composition. Studies in mice have shown that properties of *B. thetaiotaomicron* are involved in gut development from an early stage in life. When infants breastfeed, *B. thetaiotaomicron* in the intestine produces enzymes capable of digesting carbohydrates from breast milk, such as monosaccharides, oligosaccharides and polysaccharides [52]. The special enzymes produced by these bacteria suppress the host’s defense mechanisms and therefore the host is unable to prevent the digestion of glucans by the bacteria. However, a pilot study showed that oral administration of β-glucans did not influence the production of cytokines or the antimicrobial activity of leukocytes. The β-glucans, if not degraded by probiotic bacteria, have the potential to induce immune responses by stimulating the expression of pro-inflammatory cytokines by the immune cells [53]. That is, they bind to Toll-like receptor (TLR) pattern recognition (PRR) receptors, initiating a pathway that is involved in the activation mainly of macrophages and dendritic cells. When they binds to the Dectin-1 receptor, they stimulate the phosphorylation of tyrosine bound to the cytoplasmic tail of the receptor and initiate a signal transduction cascade involved in the production and release of cytokines, ROS and chemokines, and in the activation of phagocytosis [54]. Additionally, indigestion occurs if fructans and glucans are not metabolized and broken down. Furthermore, several studies show that many people who have gastrointestinal problems believe that it is due to gluten when, in fact, they have an intolerance to glucans and fructans, which is due to the absence or reduced presence of this bacterium, and so they are not metabolized [55]. It has been shown that the symptoms present in patients who thought they had gluten sensitivity were simply due to the consumption of fructans. Thus, immune suppression by bacterial enzymes is a protective mechanism against host defense, which may not be related to glycan binding to TLR and Dectin-1 receptors [56]. The benefits of this suppression for the host include uptake of oligosaccharides to meet the body’s energy needs, suppression of the inflammatory response, immune tolerance to these microorganisms and protection against allergic responses. *Bacillota* comprise another important Gram-positive phylum that plays an important role in intestinal metabolism. Some species of this phyla such as *Clostridia* spp. interact with the immune system. These species protect against inflammatory responses of the gastrointestinal tract, such as colitis and colon cancer, by causing tumor cell apoptosis [57,58]. *Faecalibacterium prausnitzii* belongs to the *Clostridium* clusters IV group, the predominant species of *Clostridia* in the intestine, and constitutes more than 5% of the total number of bacteria. It increases the production of anti-inflammatory molecules. *Clostridium butyricum* is a symbiotic bacterium and is an early colonizer in developing gut microbiota as it appears in the infant gut shortly after birth and can be beneficial for the host health. However, not all *Clostridia* spp. are helpful, some can cause infections [59,60,61,62]. Furthermore, beneficial *Clostridia* spp. can become particularly harmful in different environments with different conditions. Some strains of *C. butyricum* are also associated with illnesses in infants, such as botulism (a type of severe poisoning caused by *C. butyricum* that has toxic effects on the nervous system) and necrotizing enterocolitis (a condition causing gastrointestinal bleeding, and cell death in the mucosa) [63]. An infection of a strain of *Clostridioides difficile,* which is an opportunistic bacterium, can be life threatening. These infections are most often seen in hospitalized patients, elderly patients and in people who have experienced prolonged exposure to antibiotics [64]. Another group of bacteria found in abundance in the gastrointestinal tract from the *Actinomycetota* phyla is the *Bifidobacterium* genus, which play an important role in the host’s health. These bacteria pass through the mother’s vaginal tract to the fetus and are also present in breast milk; they are a dominant genus in the gut of the breastfed infant. Breastfeeding helps colonize the intestine with the genera *B. breve, B. bifidum* and *B. longum*. The *Bifidobacteria* protect colon cells from cancer-related mutations [65]. Among the Gram-positive bacteria found in the feces of breastfed infants are *Propionibacterium, Eubacterium* and *Bifidobacterium* (such as *B. bifidum* and *B. infantis).* Among the facultative anaerobic bacteria found in the gut, most species are from the *Lactobacillacee* family. [66]. In the colon, the communities show better stability than in the ileum [14]. Intercellular variability among microbial communities is due to internal factors (such as genetic factors, age, gender, stress and health status) or external factors. Finally, archaea are unicellular bacteria-like microorganisms that survive in an environment conducive to growth. The two species of archaea *Methanobrevibacter smithii* and *Methanosphaera stadtmanae* are present in the intestine; they are anaerobic microorganisms and require hydrogen for their functions [16]. The archaea *Methanogens* have been shown to be involved in gastrointestinal dysfunction as elevated methane levels are associated with constipation and irritable bowel syndrome (IBS) [67]. IBS is a functional disease that produces symptoms such as abdominal pain and flatulence. Increased fermentation and gas production by bacterial populations can cause the symptoms of the syndrome to appear. It has been reported that concentrations of SCFAs are increased in IBS and that they may increase the release of serotonin from the intestinal mucosa, causing an increase in intestinal permeability [12,68]. Although IBS is not a severe disease, about 10–15% of people with this disease have a reduced quality of life [69]. In contrast, inflammatory bowel diseases (IBD) are caused by chronic inflammation, which affects intestinal permeability and derives from both genetic and environmental factors (such as stress, sleep, use of antibiotics, hygiene, diet and smoking). There are two main types: ulcerative colitis and Crohn’s disease [70]. Crohn’s disease can affect the entire intestinal tract and is characterized by discontinuous involvement of different parts of the intestine, whereas ulcerative colitis is limited to the colon and rectum and is characterized by continuous inflammation of the colon [71]. Several studies have confirmed that there is a direct relationship between diet and microbiota in people with inflammatory bowel disease [72]. On the other hand, a diet rich in fruits and vegetables helps produce more SCFAs and reduces the risk of developing Crohn’s disease. Undoubtedly the human body microbiota plays an important regulatory role between humans and the environment, and many research proposals are underway to further investigate the human microbiota [73].

## 4. Effect of Probiotics and Prebiotics on the Intestinal Microbiota

**Figure 2:**
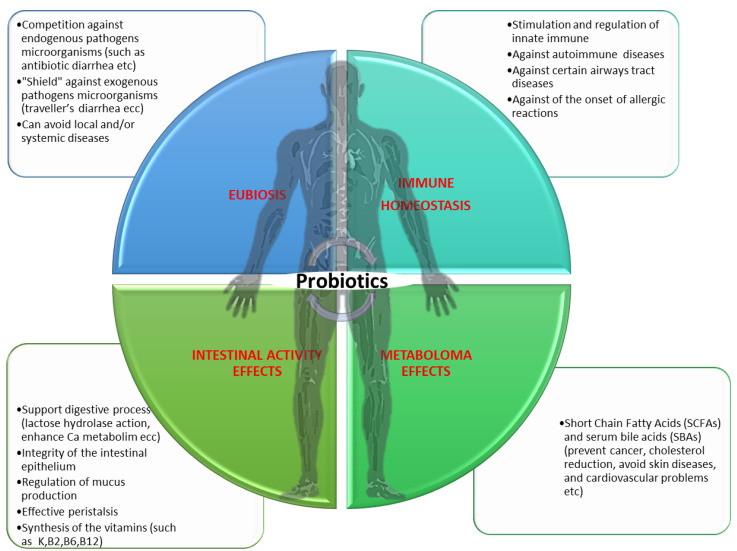
The human’s health benefits by probiotics strains. Credits: Original figure by I.A. Charitos.

As we have reported, probiotics are bacterial species that are particularly beneficial to human health (mainly including the gastrointestinal tract) when taken in sufficient quantities by individuals, whereas prebiotics are non-digestible food components such as inulin or various oligosaccharides that have been shown to stimulate beneficial bacterial populations in the colon (Figure 2) [7,8,10].

Thus, the correct functioning of the gastrointestinal system is inextricably linked to the balance of the intestinal microbiota eubiosis and to the maintenance of homeostasis and, therefore, the health of the organism. The multitude of microorganisms living in the gastrointestinal tract can positively or negatively influence the proper functioning of the digestive system, as well as other systems of the human body [31]. As we mentioned previously (see Figure 1), probiotics and prebiotics taken individually or collectively can have the beneficial effect of restoring the microbial qualitative and quantitative intestinal microbiota imbalance (dysbiosis), thus mitigating the harmful effects caused by a poor diet, use of antibiotics, infections (such as by COVID-19) and sepsis condition [74,75,76]. Oral administration is the most convenient way to give probiotic products because it can help improve the balance of the intestinal microbiota [13,14,15,16]. Foods rich in probiotic strains (such as Greek yoghurt, kefir, etc.) contain two types of microorganisms that are extremely important for the functioning of the intestine. These two types are species from *Lactobacillaceae* and *Bifidobacteriaceae* families, and they compete with pathogenic microorganisms in the intestine, such as *E. coli* and *C. perfringens* [10,77]. It has been observed that the consumption of probiotics that include these specific strains of microorganisms can lead to a significant increase in their number, and this happens in combination with a reduction in pathogenic microorganisms in the intestine [31,78]. So far, the main mechanisms of the action of probiotics include: (a) competition for dietary components as growth substrates, (b) bioconversion of sugars into fermentation products with inhibitory properties, (c) production of growth substrates, (d) direct competition from bacteriocins, (e) shielding of epithelial cells, (f) strengthening of the proper functioning of the epithelial barrier, (g) a reduction in inflammation and (h) stimulation of the innate immune response [79]. Hence, the role of probiotics in health and disease resistance is particularly important. Probiotics must be taken in sufficient quantities through functional foods to contribute to maintaining the microbiota eubiosis, protect against gastrointestinal pathogens, strengthen the immune system, control the normal levels of serum cholesterol, regulate the blood pressure, protect against the development of certain cancerous conditions, improve nutrient processing and the nutritional value of foods, promote synthesis of vitamins, enhance protein digestion, stimulate production of antimicrobial agents and aid in fighting infections [78,79,80,81]. Indeed, during our study, *H. pylori* specific probiotic microorganisms limited these negative effects by producing antimicrobial substances. This occurred due to the competitive action of specific probiotic strains against *H. pylori* and their immobilization in the stomach epithelium, such as the cultured, on cow’s milk and other substrates, symbiotic culture of strains from the *Lactobacillaceae* family and yeasts [82,83].

The metabolic intestinal activity induced by the gut microbiota can contribute to the digestion of various food compounds and the transformation of xenobiotics. On the other hand, functional food ingredients can also influence the growth and metabolic activity in these gut microbes, as well as composition species and potential functions. Functional foods can alter intestinal metabolism through the induction or inhibition of certain metabolic pathways [84,85]. The production of the highly acidic inhibitory neurotransmitter GABA (γ-amino-butyric acid) is induced by functional dietary metabolites, such as polyphenols. Indeed, prebiotic polyphenols play an important role in regulating gut microbiota. Additionally, the phenolic antioxidant compounds avenanthramides have many health benefits, such as antiatherogenic potential activity [86,87,88]. Therefore, the key to the interaction of functional foods with the intestinal microbiota lies in the fact that the components of functional foods (polyphenols, organic acids, etc.) can act as substrates in microbial metabolism, as in the case of purine alkaloids (such as caffeine) that act as purine precursors by microbial demethylation. Similarly, functional food metabolites, i.e., purine alkaloids, acted as a direct substrate in the metabolism of microbes [88,89]. The influence of functional food components on the human microbiota, such as tea phenols, showed that, in a sample of healthy volunteers, it can have an inhibitory effect on some microbial species, such as *Bacteroides* spp., *Clostridium* spp., *E. coli*, *Salmonella typhimurium*, and caffeic acid (found in *Aronia melanocarpa* and other plants*)*, which shows the highest inhibitory effect [89,90]. Olive oil processing products (such as polyphenols) have the same action by these bioactive substances as) they do with the food vector yogurt, and they can increase the concentrations of *Bacillota* phyla, *Bifidobacterium* genus, species from *Lactobacillaceae* family, and the *Clostridium perfigens* group [77,91]. *Clostridium butyricum* [92] is commonly used as a probiotic in Asian countries because it can produce metabolites (SCFAs) that benefit human health. This strain demonstrates the ability to prevent and treat antibiotic-induced diarrhea in children. It can also help to maintain the balance of *Bifidobacterium* species that may be depleted during antibiotic treatment [92]. Indeed, in mice, a strain of *C. butyricum* has been shown to protect against colitis and *C. difficile* infection [91]. Thus, probiotics modify the microbiota composition and its activity. This can also occur by means of the production of antimicrobial agents that inhibit the growth of microbes in the gut and strengthen the integrity of the intestinal barrier, resulting in a reduction in microbial translocation and modification of immune mechanisms. Indeed, the genes that allow probiotic species can be adapted to the intestinal environment, adhere to the mucosa, and interact with the immune system [31,75].

Current research has explored the relevance of a healthy, lactobacilli-dominated microbiome in preventing sexually transmitted infections and preterm labor, as well as, maintaining the quality of life for women [90,93].

Regarding the effect of probiotics on inflammatory bowel disease, controlled clinical trials conducted by researchers indicate the effective function of probiotics in combating ulcerative colitis and Crohn’s disease. More specifically, the consumption of probiotics leads to a significant increase in “friendly” bacteria living in the gut, which will dominate the intestinal microbiota, resulting in a simultaneous reduction in the unwanted ones, an increase in the short chain fatty acids that contribute to ensuring the integrity of the intestinal mucosa and an increase in the inhibition of inflammatory responses (reduction in physiological bowel inflammation) [37,78]. This implies a reduced possibility of the onset of inflammatory bowel diseases and other pathologies. The action of probiotic microorganisms against diarrhea is the most studied health effect in human clinical trials. The use of probiotics helps to prevent diarrhea that occurs in undernourished children in developing countries, but also in acute diarrhea by reducing the duration of diarrheal episodes. Probiotics spp. such as *L. rhamnosus* and *S. Boulardii* are effective both in diarrhea caused by antibiotic use and that caused by infections [94]. Furthermore, the oral administration of *L. reuteri* to mice could protect them from inflammation induced by a high-fat diet through the activation of Tregs and IL-10 production. *S. Boulardii* [95] has been shown to interfere with pathways linked to NF-κB and MAPK and attenuate the production of inflammatory cytokines [95,96]. The efficacy of *S. Boulardii* in combination with mesalamine or mesalazine versus only mesalazine has been demonstrated in patients with Crohn’s disease. Clinical results showed that recurrence of Crohn’s disease was less frequent in patients treated with both mesalazine and *S. boulardii*. In addition, a significant remission of ulcerative colitis has been shown in patients treated with *S. boulardii* [96]. It was noted that, in free-germ mice, there was a decrease in production of regulatory T lymphocytes (Tregs) in the colon [97]. When some of these mice were fed starch enriched with butyric acid, and others were fed starch enriched with propionic acid and succinic acid, there appeared to be an increase in the production of regulatory T cells (Tregs) in those fed with feed that had been enriched with butyric acid. Probiotics stimulated the maturation and activation of regulatory T lymphocytes through the production of various molecules, such as propionic acid, butyric acid, acetic acid, polysaccharide A and tryptophan D [97,98]. Probiotic species such as *F. prausnitzii* and those from the genus *Clostridium* cluster group XIVa are characterized by a high production of butyric acid. Acetic acid is produced by bacteria called acetic acid bacteria (obligate aerobes), polysaccharide A is produced by the *B. Fragilis* strain and propionic and succinic acid are product by *Bacteroidota* phyla [99,100].

Probiotic treatment has been extended to genetically modified bacteria that can express cytokines, but it can also be used as a vector for the delivery of drugs and vaccines [95]. Indeed, the intragastric administration of *Lactococcus lactis*, which secrete IL-10, reduces or prevents the dextran sulfate sodium- (DSS) induced colitis in IL-10 deficient mice. Finally, it has been demonstrated that *L. lactis* that is secreting the LcrV protein (*Y. Pseudotuberculosis*-protein) in a pseudotuberculosis mouse model has a protective role against the onset of colitis [101,102,103].

Environmental factors, hormones, tabagism and substance abuse (such as alcohol, methamphetamines, etc.) can lead to oral microbiota and/or gut dysbiosis, which can play an important role in the development of some diseases, such as autoimmune diseases like rheumatoid arthritis. *Prevotella copri* contributes to the development of rheumatoid arthritis, while *P. histicola* [104,105] suppresses it. Studies have shown that when *Bifidobacterium longum* and *L. helveticus* are taken in combination they cause a reduction in cortisol, which affects the gut/brain microbiota axis. [106]. *Companilactobacillus farciminis* is another lactic acid bacterium that is also involved in reducing intestinal permeability due to stress. Other probiotic products that contain *L. casei* are also effective in treating anxiety and depression. Giving such drinks to depressed elderly people led to an improvement in mood. All of these strains of bacteria can be characterized as psychobiotics [107].

Probiotics have an antiproliferative effect on cancer cells. The bio-mechanisms underlying the anti-cancer action are versatile and include the fight against microorganisms involved in the production and secretion of mutagens and carcinogens, the modification of the metabolism of carcinogens, the protection of DNA from oxidative damage and the regulation of the immune system. Furthermore, they have been shown to contribute to the modification of the expression of the genes involved in apoptosis and cell death, infiltration and metastasis, maintenance of cancer stem cells and cell cycle control. Further studies have shown that probiotics are involved in the modulation of signaling pathways that promote tumorigenesis [108].

As we have mentioned, prebiotics can be used to enhance the growth of specific probiotic species, promote the composition of a healthy intestinal microbiota and restore human health. Indeed, prebiotic fermentation products have important beneficial effects on human health, which include their anti-inflammatory and antiapoptotic activities and the prevention of colon cancer and colitis [109]. Specifically, *Bifidobacterium* spp. produce short-chain fatty acids by fermentation of prebiotics (such as butyrate, vital for the proper functioning and integrity of the colonic mucosa) and participate in the stimulation of the immune system [110]. Prebiotics are contained in foods rich in vegetable fibers (such as whole meal products) and are nutritional substrates such as oligofructose and inulin which favor the action of beneficial intestinal microorganisms. Prebiotics can support species from *Lactobacillaceae* and *Bifidobacteriaceae* families (which are supplied through probiotics), providing a fermentable food source for these bacteria to thrive and manifest their mechanisms of action [57,111]. Prebiotics appear to have a beneficial effect on various chronic diseases, such as idiopathic inflammatory bowel disease (such as ulcerative colitis) [112]. Administration of the specific plant fibers to people suffering from idiopathic inflammatory bowel disease is beneficial because it increases the production of beneficial metabolites for the microbiota, which in turn favor the proper functioning of the intestine, reducing either the risk of developing specific diseases or contributing to the remission of their symptoms, such as diarrhea, severe abdominal pain and feelings of malaise, which are due to inflammation present in the intestinal mucosa (Table 2) [113,114,115,116,117,118].

Thus, the beneficial short-chain fatty acids result from the colonic fermentation (breakdown of carbohydrates by anaerobic colonic bacteria). Notably, one such type of fatty acid produced through the above process is butyric acid, which activates the GRP109A receptor, which in turn is a key factor in limiting inflammation in the gut and inhibiting the activation of mast cell degranulation [113,114,115,116]. The strong anti-inflammatory properties of butyric acid and its participation in immunoregulatory and anti-inflammatory processes result in the healing of mucosal lesions, the stimulation of mucus production and the improved absorption of water and electrolytes, which are of the utmost importance for shine, for protecting the integrity of the intestinal mucosa, and for acting protectively against inflammatory bowel disease [117,118]. Finally, probiotics may improve serum folate availability that is useful during pregnancy. In fact, in a randomized clinical study conducted between the 2nd and 3rd trimester of gestation, administering probiotics based on *Bifidobacterium animalis* subsp. *lactis* (HNO19), it has been noted that it increases the serum concentration of Vitamin B9 and B12 [119].

## 5. The Process of Probiotic Encapsulation

Probiotics and their ingredients, as we have mentioned, must maintain their activity, functionality and stability under storage conditions for long periods of time, even at low temperatures. Hence, they must meet certain conditions in order to be administered or to be incorporated into a food product. However, probiotics when administered in their pure form do not exhibit these characteristics and do not appear to have good bioavailability due to their inability to penetrate cell membranes and their rapid discharge from the body. Furthermore, exposure of living organisms to antioxidants leads to antigenicity and immunogenicity issues. All these characteristics are preserved and improved by encapsulating these components in structures belonging to the nanometric scale [120,121]. Encapsulation is a process of trapping or coating one component, or, of combining components with another component. The trapped substance is usually in a liquid, gaseous or solid state. The coating medium is known as a capsule, film, or carrier. Encapsulation methods are divided into physical and chemical [122]. Physical encapsulation is in turn subcategorized into physico-chemical and physico-mechanic processes [121,123]. Chemical encapsulation includes in situ emulsion polymerization, dispersions, surface polycondensation, and suspension. Physico-chemical products include canning, solvent sublimation, solvent extraction, stratified absorption, ion gelling, complex precipitation, and supercritical fluid precipitation [124]. Finally, the physico-mechanical methods include the multi-hole centrifugal method, spray drying and coagulation, vacuum encapsulation, disc coating and electrostatic encapsulation. Of the above methods, the primary method used for microencapsulation of oral delivery systems is spray drying. This method can be applied to a wide range of materials, is compatible for encapsulation of liquids and solids, and provides particles that achieve targeted release of the entrapped component and stable capsule structure [121,125].

Microencapsulation is defined as the encapsulation process of certain materials, which are characterized as base materials, by a thin layer of polymeric material leading to the formation of micro-capsules (size of 5–5000 µm) [126]. The microencapsulation method incorporates many advantages and is therefore one of the cornerstones in the design and manufacture of oral drug delivery systems and dietary supplements [127]. In general, microencapsulation is indicated in the pharmaceutical and supplement fields because: (a) patients receive lower doses for a therapeutic effect, (b) unpleasant tastes are covered or improved in chewable capsules and powders for medicines intended for children, (c) there is a reduction in the risk of side effects, c) the mode of action of the ingredient is prolonged, (d) there is the possibility of changing the characteristics of the material when specific conditions are required, (e) the material is protected from degradation, (f) there is the possibility of a controlled and targeted distribution of the material, (g) the processing of solids and liquids is carried out in the same way and, (h) the handling of toxic substances is safe and easy [126,127].

The most common material used for encapsulating probiotics is alginate, a natural polysaccharide, which is extracted from various types of seaweed. However, this material has disadvantages, such as the possibility of producing capsids on a large scale, since the technique is time consuming. Furthermore, the microparticles obtained are porous and lack the ability to protect cells from their environment [128]. However, these defects can be overcome when the alginate is blended with other polymers, or when the polymer capsids are coated with other compounds, or when the alginate is modified using various additives [129,130]. Another microbial polysaccharide is xanthan, derived from the bacterium *Xanthomonas campestris*. Xanthan is composed of a repeating pentasaccharide unit consisting of two glucose units, two mannose units and one glucuronic acid unit. This polymer is soluble in cold water and hydrates rapidly. Another is the gellan gum derived from *Sphingomonas elodea*; it is obtained industrially by culturing the bacterium in large-scale fermentation processes. Gellan gum can produce the spherical gel structure for microencapsulation, but of its disadvantage is that it requires a high gelling temperature (80–90 °C for about one hour), which leads to thermal damage of the probiotic cells; therefore, it should be produced in a blend with xanthan gum [131]. The xanthan-gellan blend exhibits high resistance to acidic conditions and does not require such high processing temperatures that could kill bacteria. In general, after oral administration of nanoparticles, three options are considered for both probiotics and nano capsules. The nanoparticles together with the probiotics are released in the gastrointestinal tract and complete digestion with absorption takes place, then the probiotic nanocarriers are precipitated and gradually released. The encapsulated probiotics, upon release, are deposited in the various points of the gastrointestinal tract where conditions favor the survival of the probiotics. Furthermore, it is possible to form conjugates between nanocarrier residues and probiotic and prebiotic residues and thus modify their behavior in a conjugate-dependent manner (Table 3) [132,133].

Another risk is that conjugates can be transferred to other organs due to their small size. These compounds are likely to act as allergens and cause immune responses in the human body. For this reason, many studies are needed to evaluate the absorption, distribution, metabolism, and excretion of vitamin complexes not present in nanocarriers. For example, gelatin nanoparticles are formed through crosslinking and thus immune responses are induced and the antibody content increases throughout the duration of the nanoparticle’s stay in the body. In general, immune responses are more likely to be stimulated by a complex rather than a uniform structure. The nano-vehicle is resistant to digestion and therefore the probiotics are not released into the gastrointestinal tract [134].

Therefore, two versions are considered: (a) the nanoparticle and the base material are eliminated from the gastrointestinal tract and, (b) due to the nanoscale size, the nanocarrier together with the vitamin can penetrate the biological barriers of the gastrointestinal tract and thus enter the circulatory system. For this reason, toxicological tests are useful at this point. When a nanocarrier is used in the field of supplements and food, more studies are needed to determine its biodegradability, toxicological actions, and anomalous changes in material properties of the nanocarrier [135]. However, even though the application of the above methods of microencapsulation offer many possibilities for the preparation of targeted treatment systems, the entry of nanotechnology into the fields of nutrition and pharmaceuticals offers another dimension to the development of these fields [136,137,138,139]. Nanoencapsulation is defined as the technique involving the incorporation of bioactive compounds, such as vitamins, enzymes, nucleic acids, lipids, amino acids, etc. into nano-sized spheres. Nanoencapsulation technologies are divided into physical, chemical and physical/chemical [126,128]. The distribution of various components in specific body regions is directly influenced by particle size. Therefore, nanoencapsulation offers better bioavailability, possibility of controlled release and precise targeting of bioactive compounds to a greater extent than microencapsulation. In general, the main cause for a reduction in the nutritional value of ingredients is oxidation reactions [136]. Probiotics and their ingredients must meet certain conditions in order to be administered to humans or incorporated into a food product [140]. That is, they should maintain their activity, functionality, and stability under storage conditions for long periods of time, even at low temperatures. However, probiotics when administered in their pure form do not exhibit these characteristics and do not appear to have good bioavailability due to their inability to penetrate cell membranes and their rapid removal from the body. Furthermore, exposure of living organisms to antioxidants leads to antigenicity and immunogenicity issues. All these characteristics are preserved and improved by encapsulating these components in structures belonging to the nanometric scale [134]. Cell immobilization methods are generally divided into four broad categories based on the mechanism used: (a) trapping and porous matrix, where cells penetrate the porous material until they find an obstacle (other cells or the porous material within a cell culture); (b) adhesion to a solid surface through electrostatic interactions or covalent bonds; (c) aggregation by flocculation or crosslinking and (d) mechanical retention by a barrier which could be a membrane material or a microcapsule [136,140]. The greatest advantage of whole cell immobilization is that the enzymes will be active and stable for a long period of time because they are in their natural environment. The greatest disadvantage of this method is that there is a possibility of the leakage of low molecular weight enzymes from the matrix, and polyfunctional reagents used for cross linking the enzyme may denature or structurally modify the enzyme, leading to the loss of catalytic properties. In the field of nanoencapsulation of probiotics, several techniques are available at present such as: spray drying, nanoprecipitation, high-pressure homogenization, nano-emulsification, gelation, nano-complexation, agglomeration, supercritical fluid (highly compressed fluid), flow focusing, etc. Another technique is encapsulation in doughs. This technique is ideal for the encapsulation of vitamins, pharmaceutical ingredients, probiotics, essential oils and other functional ingredients, and it has many advantages over other encapsulation techniques. However, because the size of probiotic microorganisms ranges from 1–5 µm, encapsulation in nanometer scale sizes is impossible [135]. Therefore, since 1 µm equals 9-10 nm, the thickness of the capsid can exist in nanoscale dimensions and the techniques applied for µm structures are the same as those applied for nm structures, we can say that microencapsulation is an integral part of nanotechnology. The basis for the encapsulation of live probiotic organisms is immobilized cell culture technology. Encapsulation in doughs is widely used by industries since doughs are consumer friendly. Encapsulation of probiotics is usually carried out in natural and synthetic polymers [133]. When encapsulating probiotics, in nanocarriers or microcarriers, better bioavailability is observed in nanocarriers since they can easily penetrate the intestinal epithelium and enter the bloodstream [126,134].

## 6. Conclusions

In recent years, the human microbiota has been of interest and has begun to be considered as a complementary “micro-organ”, influencing many biological functions of the body. Thanks to new high-resolution technologies, access to information on the composition and functionality of the microbiota components is provided. Available clinical and experimental data have demonstrated the role of microbes in chronic non-communicable diseases. Additionally, the administration of probiotics and prebiotics to strengthen the gut microbiome is very common. The transition from a healthy to a diseased phenotype is based on a complex mechanism, which can be attributed to changes in gene and/or protein expression. The chronic diseases that have been studied the most in the last century and are related to eating habits are the following: obesity, metabolic syndrome, type 2 diabetes mellitus, cardiovascular diseases, chronic kidney disease, osteoporosis, neurodegenerative diseases and cancer. All of these are multifactorial diseases that result from interactions between different genes/proteins and environmental factors, such as bioactive food components.

However, additional studies and randomized controlled trials are needed to gain a deeper understanding of the pathogenesis of the above diseases. In summary, the consumption of prebiotics and vegetable fibers in general is important in the context of a balanced diet (such as the Mediterranean one) because the specific functional components are the necessary nutritional substrate, which, broken down by the microbial species living in the large intestine, leads to increasing concentrations of the beneficial gut microbiota for the metabolite. This results in a reduction in inflammatory cytokines and prevents the onset of chronic inflammation, thereby protecting the host’s health and reducing the risk of developing severe chronic diseases. Hence both (probiotic and prebiotic combinations) can work synergistically by increasing the number of *Bifidobacterium* spp. and *Lactobacillus* spp. in the colon.

It is generally accepted that the gut microbiota is a pioneering problem, which has been the focus of research interest nowadays due to its complexity, its involvement in several diseases and its inextricable connection with the human diet. However, the need to extensively clarify and document hitherto unrecognized and unknown aspects of the gastrointestinal microbiota, with its associations with nutrition and health, is increasingly fueling the need for further research and study. Furthermore, the current lack of sufficient research, with reliable and clear results on the subject at hand, was what motivated us to carry out the present review of primary sources. Therefore, we attempted to analyze the interconnection of the gastrointestinal microbiota with nutrition and with probiotics and prebiotics in order to investigate the relationship between them, focusing on the determination of the basic elements of a balanced daily diet that contributes to the promotion of good functioning. Thus, it is still evident at present that:

(i) diet, in addition to genetic background, functions as a critical factor in shaping the structure and composition of its intestinal microbiota;
(ii) in reference to the diet, probiotics and prebiotics balanced a nutritional model, ideal for promoting intestinal eubiosis by acting protectively for the health of the host;
(iii) an unbalanced dietary pattern (such as diet with a lack of “friendly” bacteria) causes disturbances in the composition of the intestinal microbiota (dysbiosis), which in turn is associated with various pathological conditions of the host;
(iv) diet can be customized with friendly strains in order to alleviate/prevent certain pathological conditions;
(v) some antibiotics, such as ciprofloxacin, improved the outcome of IBD patients in contrast to several experimental animal models, which showed no improvement after antibiotic administration but required bacteria colonizers for the treatment of inflammation;
(vi) more clinical trials are needed for the methods of intake with a subsequent study in detail regarding the actions of probiotics and prebiotics in shaping the intestinal microbiota and the effect on the health of the host;
(vii) an extremely interesting research proposal would be to conduct large clinical research in which the effect of particular foods with functional ingredients (such as Chios mastic) could be examined in the context of the change of the gut microbiota in some types of diseases (such as gastrointestinal diseases) in comparison with healthy subjects;
(viii) two other very important research questions are needed to determine the degree of influence of the parameters of human lifestyle acting in combination with diet, as well as to estimate the ideal composition of quantitatively and qualitatively functional gut microbiota composition associated with optimal host health.

## Figures and Tables

**Table 1:** The main probiotic strains.

	Probiotic Bacteria	
*Lactobacillaceae*	*Bifidobacteriaceae*	Other
*Lactobacillus acidophilus* *Lactobacillus johnsonii* *Lactobacillus helveticus* *Lactobacillus crispatus* *Lactobacillus gasseri* *Lacticaseibacillus casei* *Lacticaseibacillus rhamnosus* *Lactiplantibacillus plantarum subsp. plantarum* *Limosilactobacillus reuteri* *Ligilactobacillus salivarius* *Enterococcus faecium* *Leuconostoc mesenteroides*	*Bifidobacterium animalis* *Bifidobacterium breve* *Bifidobacterium infantis* *Bifidobacterium longum* *Bifidobacterium adolescentis* *Bifidobacterim lactis* *Bifidobacterim bifidum*	*Saccharomyces boulardii* *Saccharomyces cerevisiae* *Aspergillus niger* *Aspergillus oryzue* *Clostridium butyricum* *Escherichia coli*

**Table 2:** The table presents some evidence-based treatment and prevention indications for the use of probiotics and prebiotics for certain gastrointestinal disorders [118].

Probiotics and Prebiotics Evidence-Based Treatment and Prevention							
Antibiotic-Associated Diarrhea in Adults	Antibiotic-Associated Diarrhea in Children	Acute Infectious Diarrhea in Adults	Acute Infectious Diarrhea in Children *L. rhamnosus*	Reduction of Some Symptoms of Irritable Bowel Syndrome	Reduction of Symptoms Associated Maldigestion Lactose	Constipation	Hepatic Encephalopathy
*Enterococcus faecium* (10^8^ cfu, twice daily) *S. Cerevisiae* or *S. boulardii* (1 g, 3 × 10^10^ cfu on day) *L. rhamnosus* GG (10^10^–10^11^ cfu, twice a day) *L. casei* DN-114 001 (10^10^ cfu, twice a day) *L. acidophilus* CL 1285 and *L. casei* Lbc80r (5 × 10^10^ cfu, once a day)	S. cerevisiae or S. boulardii (250 mg, twice daily 3) L. rhamnosus GG (10^10^ cfu, once or twice a day) B. lactis Bb12 and S. thermophilus (10^7^ + 10^6^ cfu/g)	*E. faecium* LAB SF68 (10^8^ cfu, three times a day)	*L. rhamnosus* GG (10^10^–10^11^ cfu twice a day) *L. reuteri* ATCC 55730 (10^10^–10^11^ cfu twice a day) *L. acidophilus* and *B. infantis* (10^9^ cfu three times a day) *S. cerevisiae* or *S. boulardii* (200 mg, three times a day)	*B. infantis* 35624 (10^8^ cfu one day) *L. rhamnosus* GG (6 × 10^9^ cfu twice daily)	Yogurt with *L. bulgaricus* and S*. thermophilus* (for the digestion of the lactose of yogurt, it must not be subjected to heat treatment after pasteurization because it contains strains suitable for improving this process)	Lactulose (20–40 g daily) Oligofructose (20> g daily)	Lactulose (45–90 g daily)

**Table 3:** Some probiotic strains to add encapsulated to yogurt [133].

Probiotic Strain	Encapsulation Procedure/Material
*B. longum*	Spray drying (maltodextrin/Arabic gum)
*B. infantis*	Extrusion (gellan/xanthan gum) Emulsification (alginate)
*L. acidophilus*	Emulsification (alginate or alginate/starch) Extrusion (alginate/chitosan or Ca/alginate or Raftilose, raftiline and starch) Spray drying (maltodextrin/gum Arabic)
*B. breve*	Emulsification (fat milk and whey protein)
*B. lactis*	Extrusion (Alginate/chitosan)
*L. casei*	Emulsification (alginate) Extrusion (alginate/pectin or Alginate/chitosan
*L. rhamosus*	Emulsification (alginate)

## Data Availability

Not applicable.
